# 3D-Printed PCL Scaffolds Combined with Juglone for Skin Tissue Engineering

**DOI:** 10.3390/bioengineering9090427

**Published:** 2022-08-30

**Authors:** Musa Ayran, Akif Yahya Dirican, Elif Saatcioglu, Songul Ulag, Ali Sahin, Burak Aksu, Alexa-Maria Croitoru, Denisa Ficai, Oguzhan Gunduz, Anton Ficai

**Affiliations:** 1 Center for Nanotechnology & Biomaterials Application and Research (NBUAM), Marmara University, Istanbul 34722, Turkey; 2 Department of Metallurgical and Materials Engineering, Institute of Pure and Applied Sciences, Marmara University, Istanbul 34722, Turkey; 3 Department of Metallurgical and Materials Engineering, Faculty of Technology, Marmara University, Istanbul 34722, Turkey; 4 Department of Biochemistry, Faculty of Medicine, Marmara University, Istanbul 34722, Turkey; 5 Department of Medical Microbiology, Faculty of Medicine, Marmara University, Istanbul 34722, Turkey; 6 Department of Science and Engineering of Oxide Materials and Nanomaterials, Faculty of Applied Chemistry and Materials Science, University Politehnica of Bucharest, 1-7 Gh Polizu Street, 011061 Bucharest, Romania; 7 National Centre for Micro- and Nanomaterials, University Politehnica of Bucharest, Splaiul Independentei 313, 060042 Bucharest, Romania; 8 National Centre for Food Safety, University Politehnica of Bucharest, Splaiul Independentei 313, 060042 Bucharest, Romania; 9 Department of Inorganic Chemistry, Physical Chemistry and Electrochemistry, Faculty of Applied Chemistry and Materials Science, University Politehnica of Bucharest, 1-7 Gh Polizu Street, 011061 Bucharest, Romania; 10 Academy of Romanian Scientists, Ilfov Street 3, 50044 Bucharest, Romania

**Keywords:** Juglone, PCL, skin tissue engineering, scaffold, wound dressing, 3D printing, enhanced healing

## Abstract

Skin diseases are commonly treated with antihistamines, antibiotics, laser therapy, topical medications, local vitamins, or steroids. Since conventional treatments for wound healing (skin allografts, amnion, xenografts, etc.) have disadvantages such as antigenicity of the donor tissue, risk of infection, or lack of basement membrane, skin tissue engineering has become a popular new approach. The current study presents the design and fabrication of a new wound-dressing material by the addition of Juglone (5-hydroxy-1,4-naphthoquinone) to a 25% Polycaprolactone (PCL) scaffold. Juglone (J) is a significant allelochemical found in walnut trees and, in this study is used as a bioactive material. The effects of different amounts of J (1.25, 2.5, 5, 7.5, and 10 mg) on the biocompatibility, mechanical, chemical, thermal, morphological, and antimicrobial properties of the 3D-printed 25% PCL scaffolds were investigated. The addition of J increased the pore diameter of the 25% PCL scaffold. The maximum pore size (290.72 ± 14 µm) was observed for the highest amount of J (10 mg). The biocompatibility tests on the scaffolds demonstrated biocompatible behavior from the first day of incubation, the 25% PCL/7.5 J scaffold having the highest viability value (118%) among all of the J-loaded scaffolds. Drug release of J into phosphate buffered saline (PBS) at pH 7.4 showed that J was completely released from all 25% PCL/J scaffolds within 7 days of incubation.

## 1. Introduction

The skin is the largest organ in the human body, and it protects the body from pathogens and other antigens surrounding it. Skin also prevents moisture loss, regulates temperature, and has self-healing characteristics. Moreover, it acts like a protective barrier against ultraviolet (UV) radiation, mechanical, and thermal trauma [1]. Skin disease is one of the most frequent cause of human illness, with a large number of patients suffering from it. This morbidity is significant because it causes disfigurement, incapacity, or symptoms such as an intractable itch, which can have a significant physical and psychological influence on quality of life [2]. The treatment of skin conditions is generally based on topical therapies, such as antihistamines, antibiotics, laser therapy, local vitamins, and/or steroids [3]. Because traditional wound healing treatments (skin allografts, amnion, xenografts, and so on) have drawbacks such as donor tissue antigenicity, infection risk, and a lack of basement membrane, skin tissue engineering has become a popular technique with promising outcomes [4]. The ultimate goal of skin tissue engineering is to ensure that the normal skin anatomy regenerates, restoring both structure and function to the native skin, to supply oxygen to the wound bed, maintain appropriate moisture in the wound microenvironment, and protect against infections. Full-thickness skin regeneration with skin tissue engineering necessitates using materials that act as a temporary protective layer of the wound bed, thereby accelerating wound healing and restoring the mechanical stability and elasticity of the skin [5,6]. Three-dimensional printing plays a unique role in tissue engineering applications to produce scaffolds. These scaffolds have small pore sizes and a large surface area, which are required for perfect cellular interactions and the formation of new functional tissues. Poly(lactic-acid) (PLA) is one of the most critical upcoming biomaterials due to its advantages such as biocompatibility and excellent processability [7]. It is the most extensively researched and utilized biodegradable and renewable thermoplastic polyester with the potential to replace conventional petrochemical-based polymers [8]. Gasparotto et al. [9], developed 3D-printed scaffolds based on PLA and graphene/PLA and tested them with different cell types to assess how scaffolds influence cell behavior. The results demonstrated that the obtained scaffolds promoted cell alignment, differentiation, and myoblast fusions into multinuclear myotubes. Karabay et al. [10] evaluated a biocompatibility and toxicity assay on human dermal fibroblasts using 3D printed PLA scaffolds. The cell proliferation was demonstrated to be high even on the 18th day, these results showing that PLA scaffolds are biocompatible for a long period of time.

Various natural and/or synthetic polymers have been used as bioengineering skin grafts in wound-healing applications. For example, a 3D-bilayer-printed scaffold was designed using poly(lactic-co-glycolic acid) (PLGA) and alginate materials in order to demonstrate their use in wound-healing applications. The tissue samples demonstrated in vitro cell adhesion and proliferation, and the PLGA layer prevented bacterial invasion. Moreover, in vivo wound healing was tested using a rat model. The results showed that the scaffold reduced the wound size by 20.8% in only 4 days, and the wound was completely healed within 12 days [11].

Black walnut, butternut, hickories, and pecan are all members of the walnut family (Juglandaceae), which produces J. J is produced in the fruit and leaves and is excreted into the soil via the root system [12]. J has recently gained popularity due to studies showing that it can speed up cell proliferation, migration, and morphological recovery in skin wounds [13].

In this study, juglone was used in order to enhance wound healing as well as as an antimicrobial agent against *Staphylococcus aureus* (*S. aureus*) and *Escherichia coli* (*E. coli*), which are bacteria commonly involved in skin diseases. Three-dimesionally printed PCL was chosen as a matrix polymer. Five different amounts of J were added to this matrix polymer and tested. The purpose of this study was to find the ideal composition and to investigate the cell activity of the J-loaded scaffold for skin tissue engineering. Moreover, the studies intend to examine the influence of J addition on polymer properties such as mechanical properties, thermal stability, and antimicrobial activity. Furthermore, this research will firstly provide insight into the potential of J in the wound-healing process and its usage in skin tissue engineering.

## 2. Material and Methods

### 2.1. Materials

Polycaprolactone (PCL) and Chloroform (CHCl_3_) were supplied by Sigma Aldrich, St. Louis, MO, USA.

### 2.2. Preparation of the Solutions for 3D Printing

Firstly, 25% PCL polymer was dissolved in 10 mL of chloroform by magnetic stirring, 300 rpm, 1 h. After the dissolution, different amounts of J (1.25, 2.5, 5, 7.5, and 10 mg) were added to the 25% PCL solution and the mixture was additionally mixed for 0.5 h.

### 2.3. Fabrication of the Scaffolds with the 3D Printing Process

**Figure 1:**
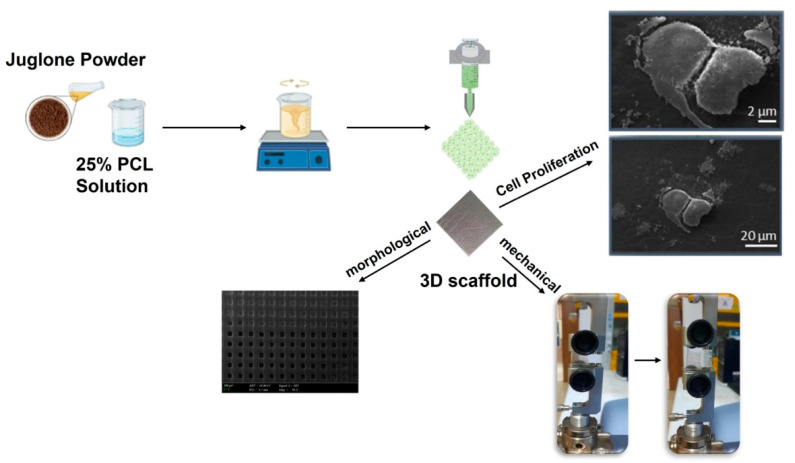
The schematic illustration of the setup.

A three-dimensional drawing software (Solidworks) was used to draw the shape of the scaffold and Slic3r was used to transform the designs into G-codes. The scaffold arrangement was planned to have square dimensions of 20 mm × 20 mm × 1 mm. The three-dimensional (3D) scaffolds were created using an extrusion 3D printer (Hyrel 3D, SDS-5 Extruder, Norcross, GA, USA) layer by layer. A 10 mL syringe with a needle with a 0.2-mm diameter was filled with polymer solutions. The printing process was conducted at a printing speed of 10 mm/s and a flow rate of 1 mL/h. The other settings were asfollows: the total layer was set to 5, the infill pattern was rectilinear, the pore size was 200 µm, and the infill degree was set to 96%. These settings were chosen during 3D printer optimization experiments. The schematic illustration of the 3D printing process is given in Figure 1.

### 2.4. Characterization of the Scaffolds

The chemical properties of the scaffolds were examined under Fourier transform infrared spectroscopy (FT-IR) over the 4000–400 cm^−1^ spectral range and 4 cm^−1^ resolution.

The surface morphologies of the scaffolds were observed with a scanning electron microscope (SEM). Before the analysis, the scaffolds were coated with gold under a vacuum, for 60 s. X-ray diffraction (XRD) analysis was used to determine the samples’ purity and crystallinity of the scaffolds. Analysis was carried out across a 2θ range between 10° and 30° at a scan speed of 2°/min using CuK radiation (λ = 1.541) at 40 kV and 30 mA. The acquired data were transformed into XRD using the OriginPro 7.0 software (OriginLab Corporation, MA, USA), and these were then assessed.

The mechanical properties of the scaffolds were determined with a uniaxial tensile testing device (EZ-LX). The force and test speed values were adjusted to 5 kN and 5 mm/min, respectively. The scaffolds were put directly into the jaws of the device and the test was repeated three times for all concentrations. The thermal properties of the scaffolds were examined with differential scanning calorimetry (DSC-6O Plus). The thermal analysis was performed in the 25–300 °C range while the heating rate was set at 25 °C/min.

The scaffolds’ antibacterial activity was tested by culturing the bacteria on Columbia Agar medium with 5% sheep blood the day before and incubating them at 35–37 °C for 24 h. The colony on the agar was removed one day later, and a 0.5 McFarland turbidity cell suspension (1–5 × 10^8^ CFU/mL) was generated in a cell densitometer device using Müller Hinton Liquid medium (MHB). The bacteria were then disseminated using a spreader on 90 mm Müller Hinton agar medium. The previously sterilized ultraviolet-light-sterilized disc samples were put on the medium. For *E. coli* (ATCC 25922) and *S. aureus* (ATCC 29213), discs containing 10 and 2 µg of ampicillin (AMP) were placed, respectively, as the control discs. In addition, all samples were put in bacteria-free MHB for contamination examination. The medium was then incubated for 24 h at 35–37 °C. Following incubation, the growth inhibition diameter around the disc was measured in millimeters.

The biocompatibility test used a human fibroblast cell line to observe the cell–material interactions. The experiment was conducted in DMEM (Dulbecco’s Modified Eagle Medium) supplemented with FBS (Fetal Bovine Serum), penicillin/streptomycin solution, and L-glutamine. The scaffolds for the cell culture test were prepared in a circular mold with a diameter of 5 mm and a thickness of 0.2 mm. They were then UV-sterilized for an overnight period. In 96-well plates, sterilized scaffolds were inserted, and fibroblast cells (5 × 10^3^/well) were plated onto the scaffolds. For a week, the scaffolds and cells were incubated in an incubator at 37 °C and 5% CO_2_. Cell viability was determined using the MTT (3-[4,5-dimethylthiazol-2-yl]-2,5-diphenyltetrazolium bromide) test, which was carried out three times. The MTT assay was used to measure the viability of the fibroblast cells on the scaffolds. After incubation (37 °C, 5% CO_2_), all media in the wells were removed, and the scaffolds were washed three times with PBS solution. A total of 90 μL of fresh medium and 10 μL of MTT solution (5 mg/mL in PBS solution) were added to the freshly cleaned scaffolds and incubated at 37 °C with a 5% CO_2_ environment for 3 h. The scaffold medium was discarded, and the scaffolds were removed to clean the wells. The scaffolds were then filled with 200 μL of DMSO to dissolve the formazan crystals, and they were placed in the incubator for 1 h. The medium was then taken from the wells, and the absorbance values were measured using a microplate reader at 540 nm.

#### In Vitro Release Studies

The release behavior of J from the 25% PCL scaffolds was performed in PBS (pH = 7.4) in a thermal shaker (BIOSAN TS-100C). Firstly, the calibration curve of the J was determined using five different J concentrations (2, 4, 6, 8, and 10 μg/mL) at a 230–300 nm wavelength range using a UV-Spectrophotometer (Shimadzu, Japan). The absorbance graph was detected using the absorbance values detected at 253 nm in the UV-spectrophotometer. The cumulative release characteristics were observed at various time intervals. Initially, 5 mg J-loaded 25% PCL scaffolds were weighed and placed in eppendorf tubes with 1 mL PBS (pH = 7.4). After each measurement, new PBS was utilized in the test.

## 3. Results and Discussions

### 3.1. Fourier Transform Infrared Spectroscopy (FTIR)

**Figure 2:**
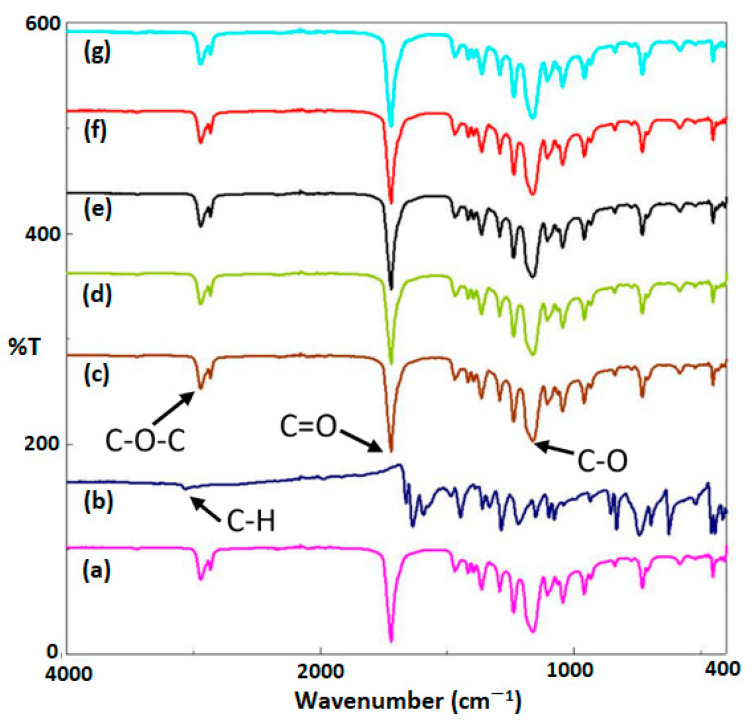
FTIR spectrums of the 25% PCL (a), pure J (b), 25% PCL/1.25 J (c), 25% PCL/2.5 J (d), 25% PCL/5 J (e), 25% PCL/7.5 J (f), and 25% PCL/10 J (g) scaffolds.

The FT-IR spectra of J and its combination with 25% PCL are comparatively demonstrated in Figure 2. The results indicated that some peaks of pure J performed similarly to various combinations of 25% PCL/J and pure 25% PCL scaffolds, stating that J was successfully loaded onto the 25% PCL scaffold. In the FTIR spectrum of 25% PCL (Figure 2a), the firm peaks were found at 2942 cm^−1^ (asymmetric), 1720 cm^−1^ (carbonyl stretching), and 1160 cm^−1^ (symmetric C-O-C stretching) cm^−1^; comparable results were investigated by other studies [14,15]. According to Figure 2b, there were strong and broad characteristic peaks of pure J at 3060 (C-H stretching), 1661 (C=O stretching), and 1288 (C-O stretching) cm^−1^. Defant et al. [16] mostly found similar peaks in the FTIR spectrum of pure J. The integration of the 25% PCL/J scaffolds exhibited a significant sharp band at 1720 cm^−1^, 1160 cm^−1^, and 2942 cm^−1^ (symmetric C-O-C stretching) which corresponds to the characteristic peak of pure J, which was initially located at 1661 cm^−1^, 1288 cm^−1^, and 3060 cm^−1^, respectively, indicating a detectable shift of the peaks toward higher wavenumbers. Additionally, the greater J content could not be readily distinguished, proving that its combination with PCL was successfully formed. As a result, FTIR spectra of 25% PCL/J demonstrated the distinctive signals of the PCL after the addition of J to the 25% PCL scaffold. In terms of the interactions between the juglone and PCL, all of the typical peaks of the 25% PCL/J underwent a considerable and unique change.

### 3.2. Morphological Characterizations of the Scaffolds

**Figure 3:**
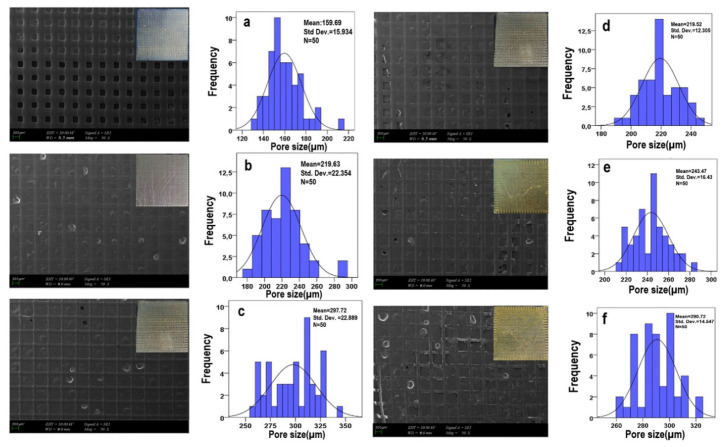
SEM images of the 25% PCL (**a**), 25% PCL/1.25 J (**b**), 25% PCL/2.5 J (**c**), 25% PCL/5 J (**d**), 25% PCL/7.5 J (**e**), and 25% PCL/10 J (**f**) scaffolds and their pore size distributions.

The morphology of the 25% PCL scaffold and its conjugations with J were determined by SEM, which depicted the images of the 25% PCL and 25% PCL/J scaffolds and the average diameter of the scaffold’s pore size in Figure 3. Figure 3 showed that the presence of J at the scaffolds significantly increased the average pore diameter, and that the concentration of J had an increasing influence on the pore size until the concentration reached 2 mg J/g PCL (the sample which contain 5 mg J). With the obtained results of the SEM images, it was evident that the 25% PCL/5 J scaffold had the minimum pore diameter (219.5 ± 12.3 µm), whereas the 25% PCL/2.5 J scaffold had the highest pore diameter (297.7 ± 22.9 µm) among the scaffolds. The spatial distribution and mutual interconnection of porosity can be specified by the standard deviation (Std). In that way, it gave us an insight into how uniform and smooth the structure was. The lowest point of std could be seen in the 25% PCL/5 J scaffold, which means that the scaffold had a more uniform structure in it if it is compared with others. Thus, it can be said that the concentration of 5 mg J was sufficient to form a uniform and beadless scaffold structure.

Three-dimensional scaffolds are generally porous structures that provide appropriate microenvironments and proper cell growth for tissue engineering [17,18]. The network structure of the pores assists in the guidance and stimulation of the formation of new tissue [19]. High-porosity materials enable the effective delivery of bio factors such as proteins and cells while also serving as suitable nutrition exchange substrates [20]. However, increasing the porosity frequently weakens the mechanical features critical for preserving the structural integrity of the biomaterial [21,22]. Regarding the pore size properties of the scaffolds, they should absorb a substantial quantity of fluid while keeping the wound bed wet, which is critical for wound healing and skin regeneration. For surgical wounds, an effective absorbent dressing/scaffold is required to minimize pad replacement during the surgical intervention as well as for chronic wounds with significant exudate production [23].

Thus, exudates are widely known for causing separation in wound tissue layers, resulting in a slower healing process. As a result, exudates must be removed from the wound site using a dressing with enough drainage capacity [24]. In this study, the 25% PCL/2.5 J scaffold was chosen to provide high absorbent properties by means of a pore structure that can generate an optimal dressing for absorbing exudates.

**Figure 4:**
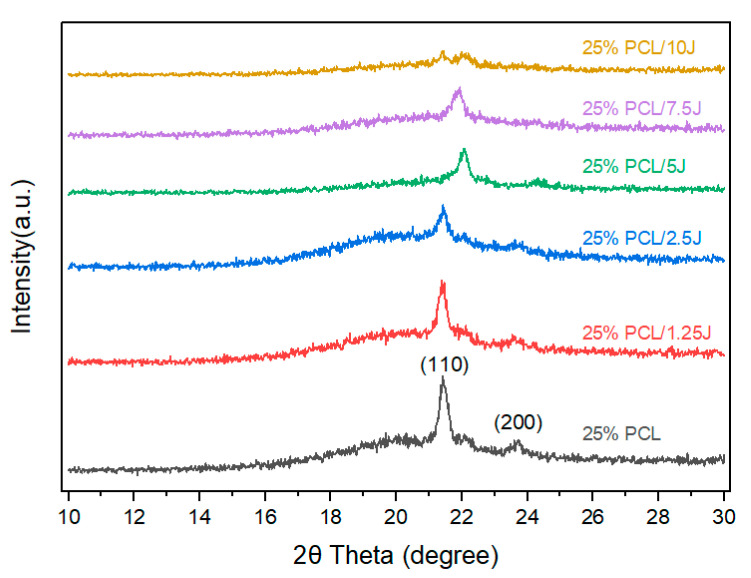
XRD pattern of the 25% PCL, 25% PCL/1.25 J, 25% PCL/2.5 J, 25% PCL/5 J, 25% PCL/7.5 J, and 25% PCL/10 J scaffolds.

Figure 4 demonstrates the XRD patterns for 25% PCL and its combination with J at different concentrations. The effect of the chains of the PCL polymer on the crystallinity of the J was inspected using XRD measurements. Pure 25% PCL exhibited two primary peaks at Bragg angles of 2θ = 21.7° and 23.8°, which were ascribed to the orthorhombic planes (110 and 200) of semi-crystalline PCL [25]. The scaffolds with a combination of J showed significant decreases in diffraction peak intensity. The process of shifting from a semicrystalline structure to an amorphous structure was demonstrated by the dramatic reduction in the diffraction peak intensity with the addition of J.

### 3.3. Mechanical Properties of the Scaffolds

The mechanical properties of the scaffolds with the addition of J in various concentrations were examined. Table 1 exhibits the scaffolds’ tensile strength, elongation, and elastic modulus values. The tensile strength of the 25% PCL scaffolds was not changed significantly by adding the J, neither was the increment of concentration, but on the other hand, the elongation of the scaffolds was increased remarkably in the presence of J which was above 200% for all tests. Furthermore, all scaffolds with J had a low elastic modulus, indicating that a small amount of stress could not induce permanent deformation, implying a ductile scaffold that was suitable for wound dressing applications in terms of flexibility, durability, and stress resistance [26,27,28]. It can be noted that the addition of 1.25 mg J to the 25% PCL scaffold increased the tensile strength by more than two times.

Compared to the other 25% PCL/J scaffolds, the addition of 10 mg J to the 25% PCL scaffold appeared to have a lower modulus and failed under low stress. Regarding the relationship between the mechanical properties and porous structure, the high pore diameter scaffolds showed relatively low mechanical characteristic properties in the scaffolds with J 2.5 mg and 10 mg. Young’s modulus (Elastic Modulus) measures the relationship between the stress and elongation of the scaffolds. The value of the Young’s modulus plays a crucial role in skin tissue, and the Young’s modulus of skin varies between 0.42 MPa and 0.85 MPa in the literature [29,30]. The 25% PCL/J (1.25, 5, and 7.5 mg) scaffolds are seemingly appropriate scaffold candidates for skin tissue because their Young’s modulus fits within the range of that of skin’s. The 25% PCL/2.5 J and 10 J scaffolds exhibited low values of Young’s modulus, enabling the scaffold to induce high deformation under elastic load in the case of skin treatment. High tensile strength values are necessary for wound treatment to preserve scaffold integrity, and high elongation at break values indicated a flexibility to operate more efficiently in wound dressing applications [31].

### 3.4. Thermal Behaviors of the Scaffolds

**Figure 5:**
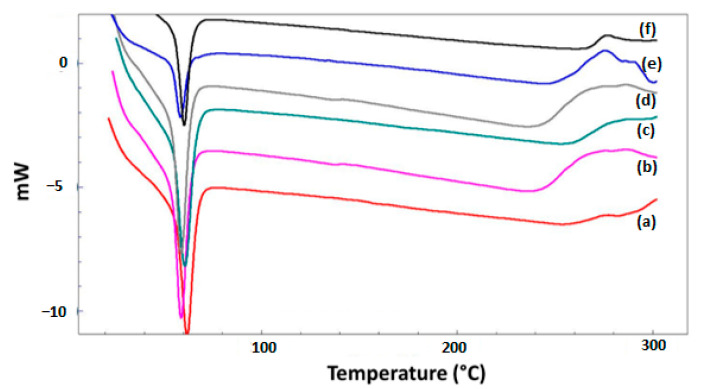
Thermal characterizations of the 25% PCL (a), 25% PCL/1.25 J (b), 25% PCL/2.5 J (c), 25% PCL/5 J (d), 5% PCL/7.5 J (e), and 25% PCL/10 J (f) scaffolds.

A proper wound dressing scaffold should be able to endure greater temperatures so that it does not disintegrate throughout the production process. Differential scanning calorimetry was used to observe the temperature transitions, and the thermograms of a single endothermic peak were acquired and are demonstrated in Figure 5. In this study, the melting temperature (T_m_) of 25% PCL was observed at 61 °C, near previously reported values [32]. It was investigated that there was no noticeable difference in T_m_ values between the scaffolds by adding J to the 25% PCL scaffold, and it was discovered that the high concentration of J altered the values slightly due to the high molecular dispersion of the 25% PCL scaffold. The presence of juglone in the scaffold proved that there was no interference between juglone and PCL in a solid state and that juglone was completely entrapped inside the polymer with no surface adsorption.

### 3.5. Antibacterial Activity Results of the J Added 3D-Printed Scaffolds

**Figure 6:**
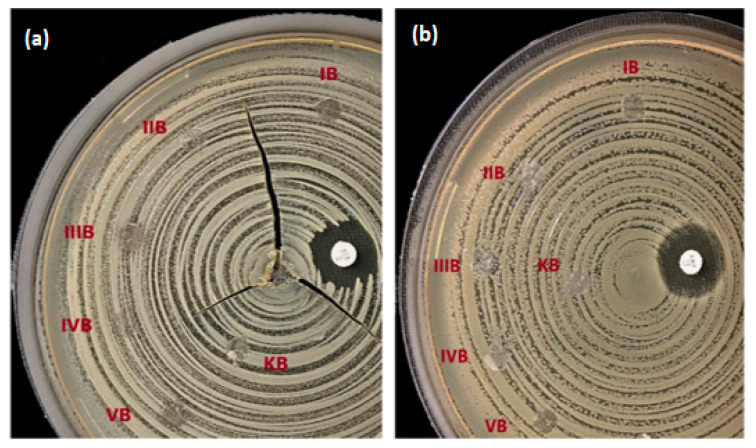
Antibacterial activity results of the scaffolds against the *S. aureus* (**a**) and *E. coli* (**b**); IB (25% PCL/1.25 J), IIB (25% PCL/2.5 J), IIIB (25% PCL/5 J), IVB (25% PCL/7.5 J), VB (25% PCL/10 J), and KB (25% PCL).

The antibacterial activity of the all scaffolds was evaluated comparatively against the gram-positive (*S. aureus*) and gram-negative (*E. coli*) strains. According to the results, all of the 25% PCL/J scaffolds showed no antibacterial action in the disc diffusion test in Figure 6. Due to the small amount of J which is solubilised and released form the patches, the antibacterial activity could not be detected against gram-negative bacteria *P. aeruginosa* and *E. coli* in the disc diffusion and agar methods. As has been previously reported in the literature, J’s antibacterial activity on *E. coli* was not observed [33]. Other research into the antibacterial effects of extracts obtained from different parts of the walnut tree has also been performed [34,35,36]. According to the findings of this research, walnut extracts were found to be less effective against gram-negative bacteria in general. The impact of commercial J and its synthetic derivatives on the antibacterial activity was stated by Clark et al. [37]. They found that gram-positive bacteria were vulnerable to commercial J, however, there was no response to gram-negative bacteria. The findings of previous investigations were consistent with our results, in that the antibacterial effect of J was not demonstrated in gram-negative bacteria.

### 3.6. Biocompatibility Properties of the Scaffolds

**Figure 7:**
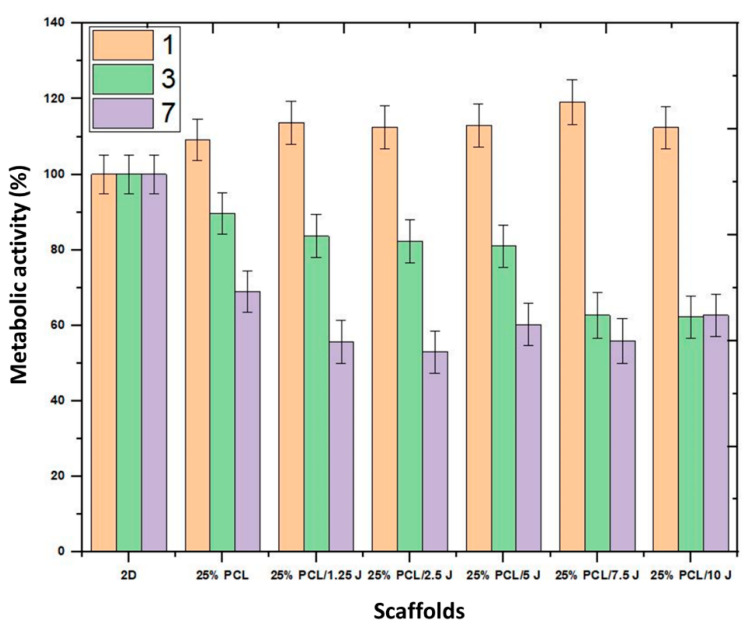
MTT assay results of the 3D J-added 25% PCL scaffolds after 1, 3, and 7 days of the culture period.

Cell proliferation tests were carried out to assess the cytocompatibility of the 25% PCL and 25% PCL/(1.25, 2.5, 5, 7.5, and 10 mg) J scaffolds using human fibroblast cells. The findings of the MTT experiment were used to determine cell compatibility, as shown in Figure 7. Following 1, 4, and 7 days of interaction with the 3D printed scaffold, the cells’ metabolic activity was measured as stated in 2D (fibroblast cell line), to which a value of 100% was assigned. Based on the content of J, the cell viability was determined to be in the 100 to 115% range within the first 24 h. The first day of data demonstrated that the cells could metabolize MTT, which improved cell survival. In the first 24 h of incubation, cell proliferation was highest (118%) in cells cultured with 25% PCL/7.5 J, whereas other J-loaded scaffolds also had high cell viability over 100% compared to the control group. On days 4 and 7, cell viability drastically dropped, and the reduction in cell viability became far more noticeable depending upon how much J was used. The long-term effect of J on cell viability may be beneficial for cancer treatment rather than therapeutic applications [38]. It was reported that J has a cytotoxic effect against various human cells, which induces apoptosis, and inhibits cell proliferation [39,40]. When comparing our results to those of previous studies, it must be pointed out that our findings showed no toxicity and more viable cells as distinct, making them favourable for the growth of fibroblast cells. Finally, the effect of J on cell proliferation was determined by the concentration and time of cell exposure to the structure, but it is essential to mention that the same behaviour can be highlighted for standard antibiotics.

**Figure 8:**
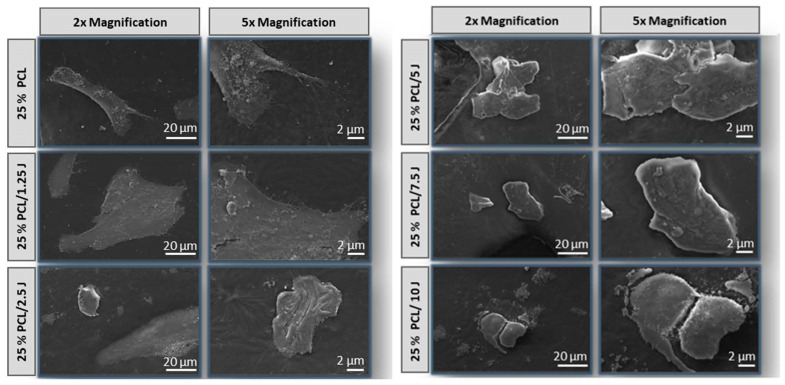
SEM images of fibroblast cell proliferation on the scaffolds after 3 days incubation. Cell growth was examined by SEM at a magnification of 2× and 5× (scale bar 2 μm and 20 μm).

Cell attachment was the first step of the cell–scaffold interactions, and it determined the ability of cells to grow and proliferate when they came into contact with the scaffold [41]. The morphological appearance of human fibroblast cells is shown at the three-day interval in Figure 8. Cellular morphology was detected in SEM images of all scaffolds, with cells spreading on the surface of scaffolds, attaching to the exterior layer of the scaffold and forming cell islets. It is commonly acknowledged that materials with a hydrophilic surface are required for cell adhesion and proliferation [42]. Due to the hydrophilic region of J [43], the fibroblast cells could interact well with the J-loaded scaffold. Our findings demonstrated that all J-loaded scaffolds supported the attachment and proliferation of human fibroblast cells, indicating that even though MTT tests highlighted the reduction in cell viability on day 3, after the release of J from the 25% PCL scaffold, 25% PCL continued to support and encourage cell adhesion and further cell proliferation.

### 3.7. In Vitro Relase Studies

**Figure 9:**
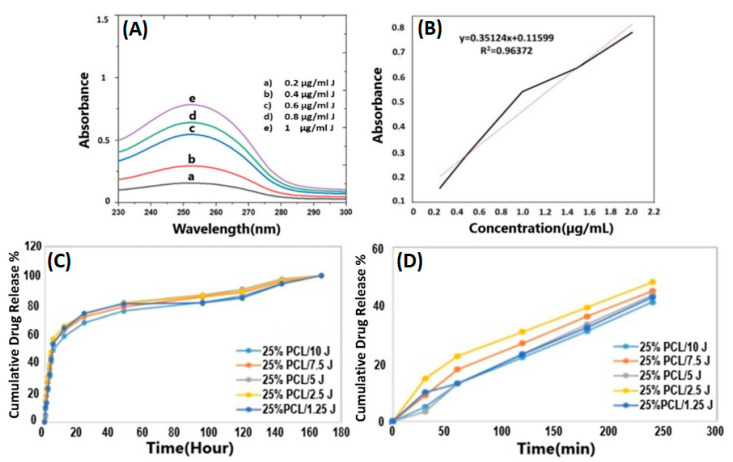
Linear calibration curve of J at different concentrations (**A**), absorbance graph obtained by calibration curve at 253 nm (**B**), full release profiles of the J (**C**), and release profiles (<4 h) of scaffolds prepared with different concentrations of J (**D**).

A modified dissolving technique in phosphate buffer solutions (PBS) at pH 7.4 was used to examine the in vitro release of J in all scaffolds. Figure 9A shows the graph used to calibrate the J using five different concentrations (ranging from 0.25 to 2 μg/mL). The absorbance graph of the J measured at 253 nm is shown in Figure 9B. J’s cumulative release from various formulations is shown in Figure 9C. Figure 9D displays the release profile of the scaffolds in under four hours prominently. All loaded scaffold formulations, as a whole, feature a biphasic release pattern characterized by an initial burst release and a later sustained release. In the release investigation, it was discovered that the drug release behaviour of the scaffolds had similar release profiles but distinct release amounts, depending on how much J was utilized in the scaffolds. By a narrow margin, 25% PCL/2.5 J was determined to exhibit the greatest quantity of J release. The 25% PCL/2.5 J scaffold showed a burst effect of over 65.7% in the first twelve hours, followed by a gradual release. Nearly 73.8% and 96.6% of the 25% PCL/2.5 J scaffold were released in the first and seventh days, respectively. J was fully released from all of the scaffolds within 168 h. In the previous study, the release patterns of the scaffolds were relatively similar, with minor variations depending on how much J was in the scaffolds [44]. Therefore, our results coherently indicated no significant differences between the J-loaded scaffolds. One reasonable phenomenon that can be attributed to why notable changes in the release profile were not observed might be the insufficient formation of a pore in the scaffold that prevented the outward movement of drug molecules positioned in the core [45].

Three-dimensional-printing technology, comparing with other techniques, such as for instance electrospinning, can lead to more consistent films, and in this case the wound dressing materials can be used even in heavily supurated wounds. In addition, the design and the production of these films can be adjusted according to needs (such as water evaporation and oxygen permeation, both characteristics being extremely important in healing as well as anti-infective behaviour) [46,47].

## 4. Conclusions

Juglone, obtained from waste walnut husks, was used to fabricate the 3D-printed 25% PCL/J scaffolds for the treatment of skin tissue. The effect of J on *S. aureus* and *E. coli* was not enough to demonstrate the potential of the natural extract for treating infections, most probably because of the low J content, but it should be enough to protect against the development of infections due to it being highly biocompatible. The addition of J exhibited low less crystallinity in comparison to the pure 25% PCL scaffolds. The tensile strength of the 25% PCL scaffold was not significantly affected by increasing the J concentration. However, as the concentration of J in the 25% PCL matrix increased, the elongation at break values rose dramatically. SEM images revealed that the addition of J enhanced the pore diameters of the 3D scaffolds. In regards to the MTT assay, it can be deduced that the cell viability increased as the amount of J increased and promoted cell adhesion and further cell differentiation up to 10 mg J content. The MTT test revealed that the scaffolds with J had no toxic effect in comparison to the control group. All J-loaded scaffolds were precisely released within seven days, and the highest quantity of J release was 25% PCL/2.5 J. Among all the scaffolds, the 25% PCL/2.5 J scaffold was selected as the most appropriate scaffold, considering its drug release behavior, pore size, and cell viability. In summary, in this study, we accomplished the fabrication of J-loaded 3D-printed 25% PCL scaffolds and indicated that the scaffolds were suitable for skin tissue applications by looking at morphological, mechanical, and cellular characteristics.

## Figures and Tables

**Table 1:** The mechanical strength values of the scaffolds.

Scaffolds	Tensile Strength (MPa)	Elongation at Break (%)	Elastic Modulus (MPa)
25% PCL	1.1 ± 0.07	97.6 ± 4.2	1.14
25% PCL/1.25 J	1.15 ± 0.28	224.7 ± 29.9	0.51
25% PCL/2.5 J	0.95 ± 0.21	263.54 ± 29.5	0.36
25% PCL/5 J	1.20 ± 0.28	262.5 ± 40.2	0.45
25% PCL/7.5 J	1.01 ± 0.25	240.50 ± 17.5	0.42
25% PCL/10 J	0.90 ± 0.12	360.45 ± 11.8	0.25

## Data Availability

The datasets created and/or analyzed during the current investigation are available upon reasonable request from the corresponding author.
